# Acceptance of Illness and Coping with Stress among Patients Undergoing Alcohol Addiction Therapy

**DOI:** 10.3390/jcm12144767

**Published:** 2023-07-19

**Authors:** Mateusz Curyło, Marlena Rynkiewicz-Andryśkiewicz, Przemysław Andryśkiewicz, Marcin Mikos, Dariusz Lusina, Jan W. Raczkowski, Olga Partyka, Monika Pajewska, Katarzyna Sygit, Marian Sygit, Elżbieta Cipora, Mateusz Kaczmarski, Łukasz Gawiński, Tomasz Banaś, Łukasz Strzępek, Grzegorz Juszczyk, Edyta Krzych-Fałta, Ewa Bandurska, Weronika Ciećko, Michał Zabojszcz, Krzysztof Zdziarski, Anna Knyszyńska, Dariusz A. Kosior, Michał Marczak, Aleksandra Czerw, Remigiusz Kozłowski

**Affiliations:** 1 Department of Internal Medicine, Rehabilitation and Physical Medicine, Medical University of Lodz, 90-647 Lodz, Poland; 2 Medical Rehabilitation Department, The Ministry of the Interior and Administration Hospital, 30-053 Krakow, Poland; 3 Department of Treatment of Alcohol Abstinence Syndromes, Independent Public Healthcare Facility in Lezajsk, 37-300 Lezajsk, Poland; 4 Faculty of Medicine and Health Sciences, Andrzej Frycz Modrzewski Krakow University, 30-705 Krakow, Poland; 5 Department of Orthopedics and Traumatology, University Hospital in Krakow, 30-688 Krakow, Poland; 6 Department of Economic and System Analyses, National Institute of Public Health NIH-National Research Institute, 00-791 Warsaw, Poland; 7 Department of Health Economics and Medical Law, Medical University of Warsaw, 02-091 Warsaw, Poland; 8 Faculty of Health Sciences, Calisia University, 62-800 Kalisz, Poland; 9 Medical Institute, Jan Grodek State University in Sanok, 38-500 Sanok, Poland; 10 Department of Management and Logistics in Health Care, Medical University of Lodz, 90-131 Lodz, Poland; 11 Department of Radiotherapy, Maria Sklodowska-Curie Institute-Oncology Centre, 31-115 Cracow, Poland; 12 Clinical Department of General and Oncological Surgery, St. Raphael Hospital Krakow, 30-693 Krakow, Poland; 13 Department of Public Health, Medical University of Warsaw, 02-091 Warsaw, Poland; 14 Department of Basic of Nursing, Faculty of Health Sciences, Medical University of Warsaw, 01-445 Warsaw, Poland; 15 Center for Competence Development, Integrated Care and e-Health, Medical University of Gdansk, 80-204 Gdansk, Poland; 16 Collegium Medicum, Jan Kochanowski University, 25-317 Kielce, Poland; 17 Subdepartment of Social Medicine and Public Health, Department of Social Medicine, Pomeranian Medical University in Szczecin, 71-210 Szczecin, Poland; 18 Department of Humanities and Occupational Therapy, Pomeranian Medical University in Szczecin, 71-103 Szczecin, Poland; 19 Mossakowski Medical Research Institute, Polish Academy of Sciences, 02-106 Warsaw, Poland; 20 Collegium of Management, WSB University in Warsaw, 03-204 Warsaw, Poland

**Keywords:** acceptance of illness, alcohol, sense of coherence, stress

## Abstract

(1) Background: Acceptance of illness is a process in which a person with an illness accepts its presence and treats it as an integral part of their life. With regard to alcoholism, acceptance of illness is one of the important elements of the healing process. (2) Methods: The study group consisted of 104 residents in an addiction treatment ward. Questionnaires SOC-29, AIS and PSS-10 were used to check levels of coherence, stress and acceptance of illness. The analysis was based on regression analysis. Patient age was analysed as a moderator of correlations between perceived indicators. Moderation analysis was based on the simple moderation model. (3) Results: The level of perceived stress correlated negatively with all areas of the sense of coherence and with acceptance of illness. All areas of the sense of coherence correlated with acceptance of illness positively. (4) Conclusions: The acceptance of illness by the patient is a factor that can be motivating for further treatment, through a positive approach to illness and strengthening the sense of control in experiencing it. The combination of strengthening behavioural, cognitive and motivational resources can be used in the treatment of people experiencing the challenges of addiction to alcohol.

## 1. Introduction

According to the data of the World Health Organization, alcohol is an identified risk factor in over 200 disease entities, and its abuse is responsible for over 5.3% of deaths annually in the world. In addition to its influence on health, alcohol has a causal relationship with many mental health disorders, which additionally translates into a worsening of the socioeconomic situation [1,2,3]. In Poland, as in many countries, there is an increasing trend in alcohol consumption. Estimates set the average annual consumption in the world at 8.7 L per person. According to the data, annual consumption in Poland is higher and amounts to 11 L per person [3]. This shows an existing problem requiring preventive action.

Apart from its physiological impact, alcohol addiction negatively affects the quality of life and the level of well-being [4,5]. Risk factors for alcoholism include genetic, psychological and environmental predispositions [6]. The results of some studies indicate the role of stress and a low level of life satisfaction in increased alcohol consumption [7]. The relationship between stress and alcohol consumption is complicated. Stress is identified as a risk factor in alcohol addiction, where, on the other hand, the motivation to consume alcohol may be the desire to reduce stress. Some studies indicate that alcohol itself acts as a stressor by activating the hypothalamic–pituitary–adrenal (HPA) axis, being the main component of the neuroendocrine stress response [8].

Acceptance of illness is a process in which a person with an illness accepts its presence and treats it as an integral part of their life [9]. According to the scientific literature, acceptance of illness constitutes an important factor affecting the level of functioning of people with chronic diseases, including alcoholism. Studies focusing on cancer patients indicate a link between the level of acceptance of illness and quality of life [10]. Similar results were obtained in studies on patients with cardiovascular diseases [11,12]. With regard to alcoholism, acceptance of illness is one of the important elements of the healing process. It is a multidimensional and active process of coping with a chronic disease [13]. Studies show that participation in the Alcoholics Anonymous (AA) program and acceptance of illness are strongly correlated with achieving permanent abstinence and improving overall mental health [14]. Acceptance of illness is also significantly correlated with the quality of life of patients with chronic diseases.

The sense of coherence (SOC) is a concept developed by Antonovsky that refers to the way an individual perceives their life as coherent, understandable and controllable [9]. Along with the increase in the level of coherence, the tendency of an individual to undertake risky behaviour decreases [15,16]. Some studies indicate that a lower level of the sense of coherence is associated with a higher risk of alcoholism [17]. One such study was conducted by Blom E et al., who examined the correlation between the sense of coherence and alcohol abuse in young adults [18]. The results suggest that people with a lower sense of coherence had a higher risk of developing alcoholism. Another study conducted by Kuntsche E et al. showed that a lower sense of coherence in adolescents was associated with a greater amount of alcohol consumed and with a greater likelihood of alcohol abuse [19].

The objective of this study was to examine the correlation between the level of acceptance of alcoholism, the sense of coherence and perceived stress among people undergoing alcohol addiction therapy.

## 2. Materials and Methods

### 2.1. Material

The sample consisted of 104 participants aged 25–70 (M = 42.78; SD = 10.57): 89 males aged 26–70 (M = 42.77; SD = 10.44) and 13 females aged 25–63 (M = 42.85; SD = 11.80) who stayed at the Department of Treatment of Alcohol Abstinence Syndromes, an Independent Public Healthcare Facility in Lezajsk, Poland. The basis for their stay in the ward was diagnosed alcohol dependence (ICD-10). All participants were undergoing treatment for alcohol dependence in the addiction treatment ward.

Three standardized tools were used in the study:

(a) Acceptance of Illness Scale (AIS)—the questionnaire is used to assess the degree of acceptance of illness by the patient. The AIS tool consists of eight statements addressing the negative effects of poor health, such as difficulties in adapting to the limitations resulting from the illness, changes in self-esteem or feelings of uselessness. The Cronbach’s alpha coefficient is 0.82, and the test–retest consistency indicator over 7 months is 0.69 [20].

(b) Orientation to Life Questionnaire (SOC-29)—the questionnaire consists of twenty-nine items divided into three subscales made of components of the sense of coherence according to Antonovsky: eleven questions checking the area of comprehensibility, ten referring to the concept of manageability and eight questions measuring the scales of meaningfulness. The Cronbach alpha coefficients of the SOC-29 research studies varying between 0.70 and 0.95 from 124 studies [21].

(c) Perceived Stress Scale (PSS-10)—the questionnaire consists of ten questions examining subjective feelings related to personal problems, related behaviours and ways of dealing with difficult situations. The overall score of the scale is the sum of all points, the theoretical distribution of which is 0–40; the higher the score is, the greater the intensity of stress experienced. The PSS-10 has good internal consistency reliability with the Cronbach’s alpha coefficient range between 0.75 to 0.91, according to studies [22].

### 2.2. Statistical Analysis

In the first step, descriptive statistics were calculated including measures of skewness and kurtosis to assess possible deviations from the normal distribution. Then, correlation analysis was performed. In the next step, perceived stress and the sense of coherence were analysed as predictors of acceptance of illness. This analysis was based on regression analysis. Finally, participant age was analysed as a moderator of correlations between perceived stress, sense of coherence and acceptance of illness. Moderation analysis was based on Hayes’ macro—Process, model 1—followed by the Johnson–Neyman procedure [23].

### 2.3. Descriptive Statistics

Table 1 presents descriptive statistics for analysed variables, i.e., mean values, standard deviations, minimum and maximum values, measures of skewness and kurtosis and Cronbach’s α reliability coefficients.

The values for measures of skewness and kurtosis did not indicate substantial deviation from the normal distribution. Therefore, subsequent analyses were based on parametric statistical methods.

## 3. Results

### 3.1. Correlation Analysis

Table 2 presents the values of Pearson’s correlation coefficients between analysed variables including participant age.

The level of perceived stress correlated negatively with all areas of the sense of coherence and with acceptance of illness. All areas of the sense of coherence correlated with acceptance of illness positively. Participant age did not correlate with the other variables.

#### Perceived Stress and the Sense of Coherence as Predictors of Acceptance of Illness

Perceived stress, the sense of coherence and participant age were analysed as predictors of health behaviours, acceptance of illness and satisfaction with life. Many statistically significant correlations between the analysed variables were detected (see Table 2). Therefore, to avoid multicollinearity, the stepwise algorithm of regression analysis was applied. Results are presented in Table 3.

General sense of coherence was the only predictor of acceptance of illness. The correlation was positive. It explained 33.2% of the acceptance of illness variance.

In addition, to verify the role of perceived stress, the variance inflation factor for the case of sense of coherence and perceived stress both were predictors of acceptance of illness in the same regression model. It was equal to VIF = 1.61, which was below the cut-off value for negative impact of multicollinearity equal to 2.5 [24]. Partial correlation between perceived stress and acceptance of illness when controlled for sense of coherence was equal to r = −0.17, *p* > 0.05. Partial correlation between sense of coherence and acceptance of illness when controlled perceived stress was equal to r = 0.37, *p* < 0.05. These additional calculations reinforce the conclusion that sense of coherence was the best predictor of acceptance of illness.

The age of the participants was not correlated with any other analysed variables. In the next step, it was analysed as a moderator of correlations between the areas of the sense of coherence and acceptance of illness. Table 4 presents values acquired for the interaction effects between participant age, the perceived level of stress and each of the sense of coherence areas from the moderation analysis.

A statistically significant interaction between comprehensibility x age was detected. Table 5 presents the regression coefficients for the correlation between comprehensibility and acceptance of illness for 22 consecutive levels of participant age expressed in terms of standardized scores acquired from the Johnson–Neyman procedure.

**Figure 1:**
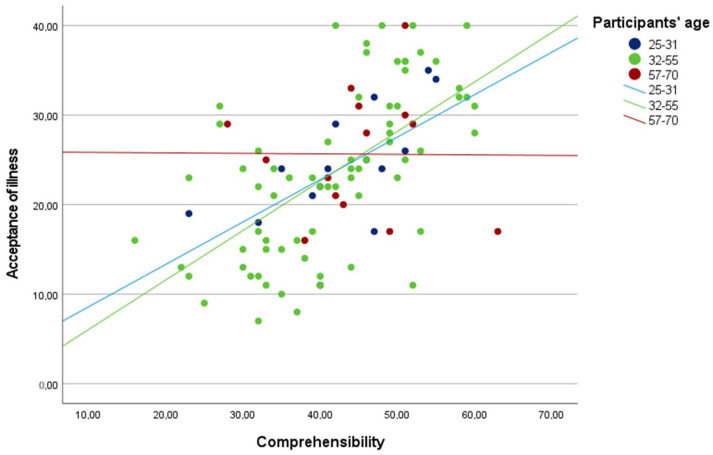
The correlation between comprehensibility and acceptance of illness depending on the participants’ age.

The positive correlation between comprehensibility and acceptance of illness was strongest for youngest participants, and then for older participants, it was weaker (see Figure 1). If participant age is higher than the standardized value equal to z = 1.46, which in the current sample translates into 58.21, the correlation between comprehensibility and acceptance of illness stops being statistically significant.

## 4. Discussion

According to the literature, the sense of coherence is associated with health behaviours. The lower the level of the sense of coherence is, the greater the tendency to undertake risky behaviours, such as excessive consumption of alcohol or other psychoactive substances [25].

In our own study, a statistically significant positive correlation was found between the sense of coherence and acceptance of illness by people addicted to alcohol (B = 0.58, t = 6.83; *p* < 0.001). In the research conducted by Chmielowiec K et al. or Bussing A et al., the obtained results also indicated a positive correlation between acceptance of illness and the sense of coherence in people undergoing alcohol addiction therapy, where people with a low level of coherence were characterized by a low level of acceptance of illness [26,27,28]. Studies indicate a positive impact of the level of acceptance of illness on the perceived quality of life of patients and increased motivation to take actions to improve their well-being [29]. However, in the study conducted by Carver CS et al., it was shown that acceptance of illness may be associated with a sense of resignation [30]. For this reason, it seems important to introduce actions that increase positive feelings.

A correlation was also found between the sense of coherence in the area of comprehensibility, acceptance of illness and the age of the patients as a moderator of the correlation. In this area, the sense of coherence and the level of acceptance of illness were higher in younger patients. This correlation was statistically significant until the mean age of 58, after which it lost its significance. According to the research results, the sense of coherence, including comprehensibility, changes during life. Some research shows that the SOC may weaken with age, and its correlation with other factors decreases with the accumulation of negative experiences. With advancing age, the strength of correlations and social role may weaken, which can lead to a decrease in the sense of self-worth and self-esteem. However, these values may affect the differences in the sense of coherence depending on the age of the respondents [31]. In the study on osteoporosis conducted by Sierakowska M. et al., in which the AIS questionnaire was used, age was also found to be correlated with the level of acceptance of illness. It was higher in the younger age groups, with an average age of 59 in the study group [32]. This area requires more in-depth analyses regarding the correlation between acceptance of illness, addiction and psychological well-being. Similar results were obtained by Pankowski D. et al. in the study on the acceptance of illness and living with illness in rheumatoid arthritis (RA) [33]. The average age of study participants was 59. The highest levels of acceptance of illness were observed in younger people; these levels decreased with age.

Our analyses showed that the sense of stress correlates negatively with all areas of the sense of coherence. Studies on the general correlation between stress and the sense of coherence indicate that the higher the level of the sense of internal coherence is, the better a person copes with stress (the level of perceived stress decreases) [34]. The study conducted by Betke K. et al. on stress and the sense of coherence among nurses showed that nurses with a high sense of coherence coped better in stressful situations. In addition, in stressful situations, persons with a high SOC less often used negative stress coping strategies, such as stimulants, especially alcohol [35]. In the study conducted by Ramchandani VA. et al., it was shown that stress increases the amount of alcohol consumed [36]. The review by Becker HC. indicates a complex correlation between alcohol addiction and stress [37]. In the initial stages of addiction, stress is a factor that enhances alcohol consumption to relieve symptoms and enhance relaxation. In contrast, long-term excessive alcohol consumption becomes a stressor, and prolonged exposure to alcohol leads to fundamental changes in the brain reward systems and neuroendocrine stress systems, which disrupts the physiological control systems for ethanol consumption [37]. The results show that a high SOC is correlated with reduced alcohol consumption according to some studies conducted in younger age groups [38]. Research indicates that implementing actions aimed at using internal resources and strengthening the SOC can have a positive effect on health and enhance overall life experience. The concept of salutogenesis is used in building tools for coping with stress in the psychoeducation process [39,40].

## 5. Limitations

The main limitation of this study was the fact that data were collected at one point in time, which does not make it possible to compare changes in the value of indicators over time. The objective of the study, however, was not to present changes over time, but to check the level of acceptance of illness among people undergoing alcohol addiction therapy. The size of the study group may also constitute a limitation in making subgroup comparisons. Differences in withdrawal stages were also not considered in the study. For this reason, it is possible that relationships that would otherwise be identifiable may not be detected, and a small sample increases the chance of assuming a false premise as true.

## 6. Conclusions

The concept of the sense of coherence may have significant potential to be used in building resources necessary to protect people against the effects of negative events by strengthening the sense of self-efficacy and endurance. The acceptance of illness by the patient is a factor that can be motivating for further treatment, through a positive approach to illness and strengthening the sense of control in experiencing it. The use of salutogenesis in the treatment of alcoholism may contribute to building internal resources, including the creation of mechanisms that reduce tension and stress in the face of negative experiences such as addiction.

## Figures and Tables

**Table 1:** Descriptive statistics for analysed variables.

Variables	*M*	*SD*	*min*	*max*	*S*	*K*	α
Perceived stress	21.79	6.92	4	38	−0.24	−0.29	0.87
Comprehensibility	42.20	9.97	16	63	−0.27	−0.44	0.78
Manageability	45.39	9.06	26	65	−0.05	−0.40	0.75
Meaningfulness	39.90	8.42	13	56	−0.69	0.41	0.79
Sense of coherence	127.50	23.13	63	176	−0.25	−0.30	0.89
Acceptance of illness	24.05	8.62	7	40	0.07	−0.82	0.87

*M*—mean value; *SD*—standard deviation; *min*—minimum value; *max*—maximum value; *S*—measure of skewness; *K*—measure of kurtosis; α—Cronbach’s alpha reliability coefficient.

**Table 2:** Correlation coefficients between analysed variables.

Variables	Perceived Stress	Comprehensibility	Manageability	Meaningfulness	Sense of Coherence	Acceptance of Illness
Perceived stress	-	-	-	-	-	-
Comprehensibility	−0.576 **	-	-	-	-	-
Manageability	−0.548 **	0.666 **	-	-	-	-
Meaningfulness	−0.419 **	0.485 **	0.528 **	-	-	-
Sense of coherence	−0.616 **	0.869 **	0.871 **	0.780 **	-	-
Acceptance of illness	−0.439 **	0.502 **	0.479 **	0.346 **	0.530 **	-
Age	−0.102	0.024	0.077	0.031	0.051	0.005

** *p* < 0.01.

**Table 3:** Analysis of perceived stress and the sense of coherence as predictors of acceptance of illness.

Dependent Variable	Predictors	*B*	*t*	*p*	Δ*R*^2^
Acceptance of illness	Sense of coherence	0.58	6.83	0.001	0.33

*B*—standardized regression coefficient; *t*—value of test for predictor’s significance; *p*—statistical significance; Δ*R*^2^—change in determination coefficient when the predictor added to a model.

**Table 4:** Interaction effects between participant age, the perceived level of stress and each of the sense of coherence areas.

Interaction Effects	*B*	*t*	*p*
Perceived stress x Age	−0.02	−0.16	0.874
Comprehensibility x Age	−0.21	−2.19	0.031
Manageability x Age	−0.06	−0.64	0.522
Meaningfulness x Age	0.01	0.02	0.985
Sense of coherence x Age	−0.09	−1.00	0.321

*B*—standardized regression coefficient; *t*—test for statistical significance of regression coefficient; *p*—statistical significance.

**Table 5:** The correlation between comprehensibility and acceptance of illness depending on participant age.

*z*	*B*	*t*	*p*
−1.68	0.93	5.01	0.001
−1.47	0.89	5.27	0.001
−1.26	0.84	5.57	0.001
−1.04	0.80	5.91	0.001
−0.83	0.76	6.28	0.001
−0.62	0.72	6.66	0.001
−0.41	0.67	6.99	0.001
−0.19	0.63	7.15	0.001
0.02	0.59	7.01	0.001
0.23	0.54	6.49	0.001
0.45	0.50	5.67	0.001
0.66	0.46	4.73	0.001
0.87	0.42	3.84	0.001
1.09	0.37	3.06	0.003
1.30	0.33	2.41	0.018
1.46	0.30	1.99	0.050
1.51	0.29	1.88	0.064
1.72	0.24	1.44	0.154
1.94	0.20	1.07	0.286
2.15	0.16	0.77	0.444
2.36	0.11	0.51	0.609
2.58	0.07	0.30	0.768

*z*—participant age standardized; *B*—standardized regression coefficient; *t*—value of test for predictor’s significance; *p*—statistical significance.

## Data Availability

The data analysed during the current study are available at the authors.
